# Inhibition of visceral nociceptors

**DOI:** 10.3389/fphar.2014.00072

**Published:** 2014-04-14

**Authors:** David E. Reed, L. Ashley Blackshaw

**Affiliations:** Neurogastroenterolohy Group, Centre for Digestive Diseases, Blizard Institute, Queen Mary University of LondonLondon, UK

**Keywords:** nociceptors, inhibition, visceral pain, enteroendocrine cells, intestinal mucosa

Abdominal pain is one of the most frequent reasons for consulting a doctor. Despite it being a common clinical presentation, abdominal pain remains a difficult entity to treat. This is a result of multiple factors including time course (i.e., acute vs. chronic), etiology (e.g., inflammatory vs. post-inflammatory), and stimulus (i.e., mechanical vs. chemical). About 10% of the population suffer from chronic visceral pain in the form of irritable bowel syndrome (IBS), many of which go undiagnosed. They are hypersensitive to contraction and distension of the gut (Barbara et al., 2011; Keszthelyi et al., 2012), but the pathophysiology of pain is poorly understood. While the intestinal epithelium is a site of high metabolic activity, including digestion/absorption, secretion, hormone release, and immune interactions, it is generally not viewed as a site of pain modulation. However, there are numerous factors at the level of the epithelium capable of modulating pain. This article will highlight the potential role of these factors in nociceptive signaling to identify new therapeutic targets.

## Nociceptive innervation of the gut

Like in other regions of the body and internal organs, the identification of which gastrointestinal primary afferent neurons transmit signals giving rise to pain relies on classification according to their adequate stimuli. It is well-known that cutting and burning of the gut is not necessarily perceived as painful, but generation of intense force by distension or contraction normally is (Cervero, 1994). In the diseased gut less intense forces are required (Coutinho et al., 1996). Therefore, a gut nociceptor is defined for the purposes of this article as a primary afferent fiber that has a high threshold to mechanical stimuli in the healthy gut. They usually also respond directly to inflammatory mediators (Blackshaw et al., 2007). A number of investigations over the last five decades have shown these to innervate blood vessels either within or outside the gut wall (Bessou and Perl, 1966; Blumberg et al., 1983; Song et al., 2009). They are therefore a form of vascular endings, whereas low-threshold afferents innervate the muscle layers of the gut wall or the villi of the mucosa (Brookes et al., 2013). An exception to this rule may well be esophageal afferents which may transmit pain directly from the squamous epithelium in response to acid (which may reflux from the stomach) (Bhat and Bielefeldt, 2006). In the abdominal viscera, it makes sense to place nociceptive endings on blood vessels, since these are less likely to be exposed to mechanical force than those in smooth muscle, and would serve an alarm function for impending dangerous events like rupture or bleeding of the gut. In general, nociceptive afferents innervate the gut via the splanchnic nerves, or in the esophagus via the thoracic sympathetic nerves. There are also sub-populations that innervate via the pelvic and vagal parasympathetic pathways in the rectum and esophagus, respectively.

Whether or not visceral nociceptors differ from those in the rest of the body is controversial, but there are accounts of differing gene expression between the two systems, e.g., Brierley et al. (2008). Another important issue is how nociceptors change in disease states, which may change the way we define them. It is clear from a number of studies that their mechanical thresholds are substantially reduced during inflammation and after healing, so that they respond within the physiological range of stimuli (Jones et al., 2005). In fact Hughes et al found that only nociceptors became markedly sensitized after recovery from inflammation, in both pelvic and splanchnic pathways, whereas other types of afferent fibers were affected little or not at all (Hughes et al., 2009).

The central endings of visceral afferents are most commonly found in the dorsal horn of the spinal cord, where they synapse on projection neurons that send axons to pain processing areas of the brain (Honoré et al., 2002). The dorsal horn is a site of major modulation of pain signals which can override or exacerbate the peripheral signal. Another major site of modulation that is emerging is at the site of signal generation in peripheral endings by a number of endogenous factors. These may be derived from enterocytes, the most numerous cells in the epithelium, or from more specialized cells such as enteroendocrine or immune cells. The examples given below are the best we can find currently and represent derivatives from these three cell types, respectively.

## Cyclic-guanosine-3′,5′-monophosphate (cGMP)

Recently it has been demonstrated that stimulation of guanylate-cyclase C (GC-C) on epithelial cells causes release of cGMP (Blackshaw and Brierley, 2013). This extracellular cGMP inhibits nociceptors in the colon and rectum (Castro et al., 2013; Feng et al., 2013). This is believed to be a mechanism by which linaclotide, a GC-C agonist, alleviates pain in patients with IBS constipation subtype. Demonstration of this epithelial-afferent anti-nociceptive signaling raises the possibility that other mediators released from epithelial cells can inhibit painful signals arising from the gut.

## Glucagon-like peptide-1 (GLP-1)

GLP-1 is an incretin located within enteroendocrine L-cells of the intestine that is released upon food intake (Punjabi et al., 2011). It plays an important role in postprandial glucose homeostasis and can alter gastrointestinal (GI) motility (Näslund et al., 1999; Hellström et al., 2008; Edholm et al., 2009; Punjabi et al., 2011). Interestingly, in a phase II trial of IBS patients the GLP-1 analog ROSE-010 reduced acute exacerbations of abdominal pain compared to placebo (Hellström et al., 2009). Whether this analgesic effect was a primary effect on afferent fibers innervating the gut or secondary to a reduction in dysmotility could not be determined. It has been shown that GLP-1 can directly activate vagal afferents within the upper GI tract (Gaisano et al., 2010). However, the expression of GLP-1 is greatest in the distal GI tract. Taken together, this raises an intriguing possibility that release of GLP-1 from L-cells in the distal gut may inhibit spinal afferents responsible for nociceptive transmission. Many treatments for type 2 diabetes are aimed at augmentation of the action of GLP-1 on the endocrine pancreas to boost insulin release. It would be interesting to determine if these treatments have a parallel effect on visceral pain, although this may be small since the effect of GLP-1 on afferent fibers is probably a paracrine effect whereas GLP-1 mimetics augment endocrine actions.

## Somatostatin

There are other epithelial derived mediators with more prominent roles in the upper GI tract that may be capable of inhibiting visceral pain. Somatostatin is found in a variety of cell types in the gut including D-cells in the gut mucosa (Patel, 1999). Its role is largely inhibitory of a number of physiological functions within the GI tract including secretion and motility (Patel, 1999). There is also evidence that it can inhibit visceral perception. Somatostatin receptor agonists reduced afferent firing in “wide-dynamic range” fibers, which would be expected to transmit noxious stimuli (Booth et al., 2001). Jejunal afferents in knockout mice lacking the somatostatin receptor *sst*2 had augmented responses to both low and high threshold distension as well as chemical stimulation suggesting a tonic inhibitory role of somatostatin in visceral sensitivity (Rong et al., 2007). Conversely, in recordings from pelvic afferents in rats, the somatostatin receptor agonist octreotide appeared to reduce visceral pain via a central mechanism rather than at the peripheral site (Su et al., 2001). Interestingly, octreotide inhibited sensation to rectal balloon distension in both healthy volunteers and IBS patients (Hasler et al., 1993, 1994). The investigators believed this was likely due to a peripheral action of octreotide as previous work strongly suggested that octreotide peripherally inhibited cerebral and spinal electrical potentials after electrical stimulation of the rectum (Chey et al., 1995; Schwetz et al., 2004). These contrasting findings suggest that somatostatin may signal to specific afferent subtypes and/or there are species differences. Therefore, further investigations of an inhibitory role of somatostatin on visceral nociception in the distal gut are needed.

## Galanin

Galanin is an important neuromodulator in the enteric nervous system, which acts on three types of G-protein coupled receptor—GAL1, 2, and 3, which are inhibitory, excitatory, and inhibitory respectively. GAL1 and 2 have corresponding effects on mechanosensitivity of vagal afferents at low concentrations (Page et al., 2007), and may indeed have an effect endogenously, but we have only preliminary unpublished data indicating an inhibitory effect of galanin on spinal afferents. It is clear that galanin may have potent central effects on pain processing, especially in neuropathic and inflammatory pain (Wynick et al., 2001), but this peripheral action in the gut is unexplored.

## Opioids

T-lymphocytes contain and release beta-endorphin, which activates the mu opioid receptor. This is considered to be an important mechanism of endogenous pain relief, and part of the action of exogenous opioids given therapeutically. We recently showed this is also the case for the gut, where these cells migrate into the tissue and inhibit the response of nociceptors to mechanical stimuli (Hughes et al., 2013b). However, T-cells also release interleukins and other cytokines that can augment the sensitivity of nociceptors or even activate them directly, so there is a balance of excitatory and inhibitory immune modulation of peripheral nociceptive signaling. It is not known if stimuli to the gut in health or disease can preferentially evoke release of opioids or other mediators from white cells, but this would provide a convenient means of pain suppression during normal digestion.

In addition to mu receptors, visceral afferents also express kappa opioid receptors, for which the natural ligand is dynorphin, although it is unclear if endogenous dynorphin plays a role in peripheral pain modulation. However, it has been known for some time that kappa opioid receptor agonists reduce activation of colorectal afferents by distension, and correspondingly pain behaviors evoked in conscious animals (Gebhart et al., 2000). Recent data indicates that visceral nociceptors increase their expression of kappa receptors in a model of chronic visceral hypersensitivity (Hughes et al., 2013a), and correspondingly their inhibition by kappa agonists. This probably underlies the clinical efficacy of kappa ligands on pain in moderate to severe IBS (Mangel and Hicks, 2012).

## Conclusions

In addition to the scope for many interventions that could reduce visceral pain in the clinic targeted at inhibitory mechanisms, there are also many sources of endogenous inhibitors, only some of which have been explored. It is clear nutrients, microbiota and other immunomodulatory influences may impact on the behavior of nociceptors, for example by releasing enteroendocrine mediators or immune-derived mediators. In the case of microbiota, there is also the possibility of direct actions of microbial products on sensory endings, as has already been shown to occur elsewhere in the nervous system (Hsiao et al., 2013).

### Conflict of interest statement

The authors declare that the research was conducted in the absence of any commercial or financial relationships that could be construed as a potential conflict of interest.
